# Non-Stimulated, Agonist-Stimulated and Store-Operated Ca^2+^ Influx in MDA-MB-468 Breast Cancer Cells and the Effect of EGF-Induced EMT on Calcium Entry

**DOI:** 10.1371/journal.pone.0036923

**Published:** 2012-05-30

**Authors:** Felicity M. Davis, Amelia A. Peters, Desma M. Grice, Peter J. Cabot, Marie-Odile Parat, Sarah J. Roberts-Thomson, Gregory R. Monteith

**Affiliations:** School of Pharmacy, The University of Queensland, Brisbane, Queensland, Australia; University of Queensland, Australia

## Abstract

In addition to their well-defined roles in replenishing depleted endoplasmic reticulum (ER) Ca^2+^ reserves, molecular components of the store-operated Ca^2+^ entry pathway regulate breast cancer metastasis. A process implicated in cancer metastasis that describes the conversion to a more invasive phenotype is epithelial-mesenchymal transition (EMT). In this study we show that EGF-induced EMT in MDA-MB-468 breast cancer cells is associated with a reduction in agonist-stimulated and store-operated Ca^2+^ influx, and that MDA-MB-468 cells prior to EMT induction have a high level of non-stimulated Ca^2+^ influx. The potential roles for specific Ca^2+^ channels in these pathways were assessed by siRNA-mediated silencing of ORAI1 and transient receptor potential canonical type 1 (TRPC1) channels in MDA-MB-468 breast cancer cells. Non-stimulated, agonist-stimulated and store-operated Ca^2+^ influx were significantly inhibited with ORAI1 silencing. TRPC1 knockdown attenuated non-stimulated Ca^2+^ influx in a manner dependent on Ca^2+^ influx via ORAI1. TRPC1 silencing was also associated with reduced ERK1/2 phosphorylation and changes in the rate of Ca^2+^ release from the ER associated with the inhibition of the sarco/endoplasmic reticulum Ca^2+^-ATPase (time to peak [Ca^2+^]_CYT_ = 188.7±34.6 s (TRPC1 siRNA) versus 124.0±9.5 s (non-targeting siRNA); *P*<0.05). These studies indicate that EMT in MDA-MB-468 breast cancer cells is associated with a pronounced remodeling of Ca^2+^ influx, which may be due to altered ORAI1 and/or TRPC1 channel function. Our findings also suggest that TRPC1 channels in MDA-MB-468 cells contribute to ORAI1-mediated Ca^2+^ influx in non-stimulated cells.

## Introduction

Plasma membrane Ca^2+^ channels, including voltage-gated, transient receptor potential (TRP), and store-operated Ca^2+^ channels, regulate Ca^2+^ influx in a highly controlled and cell type-dependent manner to regulate specific cellular processes. For example rapid Ca^2+^ influx through voltage-gated Ca^2+^ channels at the neurological synapse regulates the exocytosis of neurotransmitters from presynaptic neurons [1]. An influx pathway increasingly recognized as a regulator of intracellular signaling processes in epithelial cells is store-operated Ca^2+^ entry [2]–[4]. The molecular components for this influx pathway, which is responsible for replenishing endoplasmic reticulum (ER) Ca^2+^ reserves following Ca^2+^ store depletion, include the store-operated Ca^2+^ channel pore-forming subunits ORAI1, ORAI2 and ORAI3 [5]–[7] and their classical activators the ER Ca^2+^ sensors STIM1 and STIM2 [8], [9]. Activation of ORAI1-mediated Ca^2+^ influx by other proteins has also been identified; this includes regulation by pre-STIM2 [10] and SPCA2 [11], the latter of which is a Ca^2+^ store-independent mechanism important in some breast cancers. Although ORAI proteins are now recognized as the central components of store-operated Ca^2+^ entry, the canonical-type TRP channels (TRPCs), in particular TRPC1, are also reported to interact with STIM proteins and in some cell types appear to regulate agonist-stimulated and store-operated Ca^2+^ entry [4], [12]. Mechanisms governing the pathways involved in replenishing ER Ca^2+^ reserves, such as the ion channels involved and their activation, may therefore be context-dependent and vary between cell types.

In breast cancer cells, ORAI1 regulates processes important for carcinogenesis and is enriched in some breast cancer cell lines relative to non-tumorigenic breast epithelial cells [13]. Knockdown of ORAI1 is anti-proliferative in MCF7 breast cancer cells and is associated with reduced activity of extracellular signal-regulated kinase (ERK1/2) [11]. Furthermore, inhibition of ORAI1 in invasive MDA-MB-231 breast cancer cells reduces serum-induced migration *in vitro* and metastasis formation *in vivo* [14].

A process implicated in breast cancer metastasis is the transition from an epithelial state to a more invasive mesenchymal phenotype, termed epithelial-mesenchymal transition (EMT) [15]. This phenotypic switch involves changes in cell morphology, expression of the type III intermediate filament vimentin, and the production and secretion of proteases at the leading edge to facilitate invasion [15], [16].

A cellular conversion comparable to EMT that is particularly important in atherosclerotic vascular disease, involves the transformation of vascular smooth muscle cells from a contractile (quiescent) to a synthetic (proliferative) phenotype [17]. This change is associated with a remodeling of Ca^2+^ influx pathways, including elevated store-operated Ca^2+^ entry [18]. In breast cancer cells, Ca^2+^ signaling requirements are also likely to differ as cells undergo EMT and transition from an epithelial phenotype to a mesenchymal state. Indeed, EGF-induced EMT in MDA-MB-468 breast cancer cells is associated with a remodeling of purinergic receptor-mediated Ca^2+^ signaling [19] and transforming growth factor β (TGFβ)-induced EMT in MCF7 breast cancer cells has been reported to be associated with increased store-operated Ca^2+^ influx [20]. Here we sought to assess changes in store-operated Ca^2+^ entry in a well characterized model of EMT mediated by EGF in MDA-MB-468 breast cancer cells. A greater understanding of the changes in Ca^2+^ influx associated with EMT in breast cancer cells may help identify novel therapeutic targets for the control of breast cancer metastasis.

## Materials and Methods

### Cell culture and reagents

MDA-MB-468 [19] and MDA-MB-231 [21] human breast cancer cells were cultured in DMEM (D6546, Sigma Aldrich) containing 10% fetal bovine serum (FBS), 4 mM L-glutamine, 100 U/mL penicillin and 100 µg/mL streptomycin. Cells were maintained in a humidified 37°C incubator with 5% CO_2_ and were routinely screened for mycoplasma contamination (MycoAlert, Lonza). Cyclopiazonic acid (CPA), trypsin and ATP were purchased from Sigma Aldrich. Fluo-4 AM and BAPTA were purchased from Invitrogen. TaqMan Gene Expression Assays (Applied Biosystems) included: human Twist1 (Hs00361186_m1), Snail (Hs00195591 _m1), fibronectin (Hs01549976_m1), vimentin (Hs00185584_m1), ORAI1 (Hs00385627_m1) and TRPC1 (Hs01553152_m1). Dharmacon On-TARGET *plus* SMARTpool™ siRNAs were used at a final concentration of 100 nM: non-targeting (D-001810-10-05), TRPC1 (L-004191-00-0005) and ORAI1 (L-014998-00-0005). The following Cell Signaling antibodies were used: mouse monoclonal anti-phospho-ERK1/2 (Thr202/Tyr204; 9106) at 1∶2000 and rabbit polyclonal anti-ERK1/2 (9102) at 1∶1000, incubated overnight; and from BioRad, anti-mouse horseradish peroxidase-conjugated and anti-rabbit horseradish peroxidase-conjugated secondary antibodies (1∶10,000).

### Measurement of intracellular calcium

**Figure 1:**
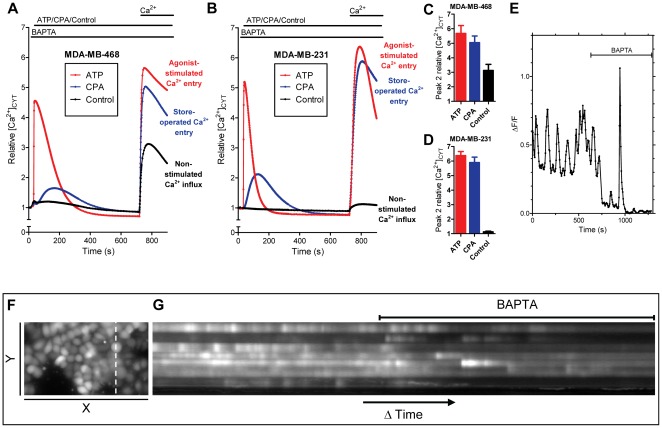
Assessment of cytosolic Ca^2+^ signaling in MDA-MB-468 and MDA-MB-231 breast cancer cell lines. Average ATP-stimulated Ca^2+^ entry response (100 µM ATP), store-operated Ca^2+^ entry response (10 µM CPA) and non-stimulated Ca^2+^ influx (DMSO control) were assessed in **A**) MDA-MB-468 and **B**) MDA-MB-231 cells. Quantitation of peak relative Ca^2+^ influx (peak 2) in **C**) MDA-MB-468 and **D**) MDA-MD-231 cells; shown as average ± S.D. (n = 9 for MDA-MB-468 & n = 4 for MDA-MB-231 cells). **E**) MDA-MB-468 cells exhibit spontaneous and asynchronous Ca^2+^ oscillations that are attenuated by extracellular Ca^2+^ chelation (BAPTA). **F**) Still image showing the Y-plane section and **G**) the Y-plane projection through time. See also Movie S1. Representative of five movies from three independent experiments.

**Figure 2:**
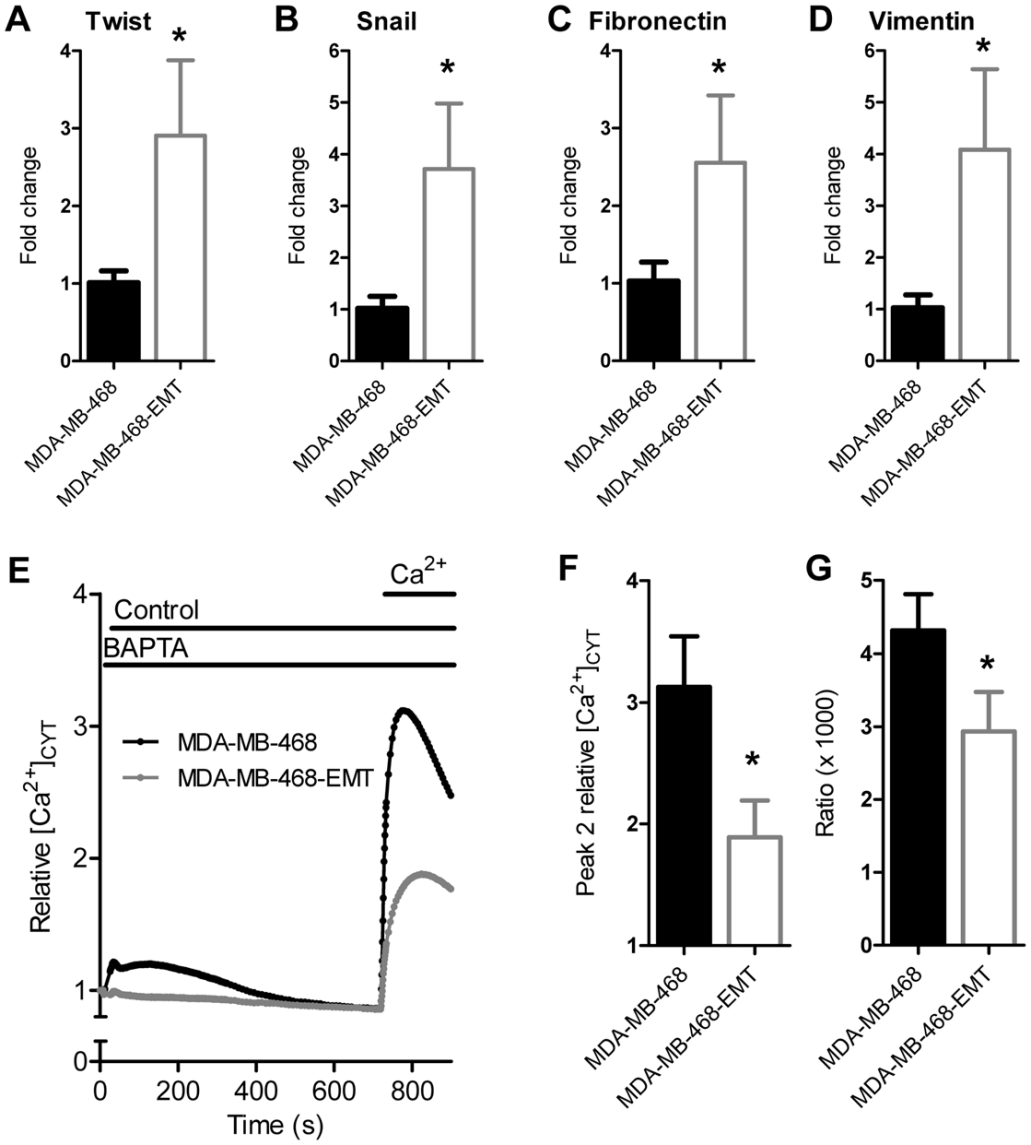
Non-stimulated Ca^2+^ influx in MDA-MB-468 cells induced to undergo EMT with EGF. MDA-MB-468 breast cancer cells stimulated with EGF to induce EMT (MDA-MB-468-EMT) have elevated transcription of the mesenchymal markers **A**) Twist, **B**) Snail, **C**) fibronectin and **D**) vimentin. MDA-MB-468-EMT cells show reduced non-stimulated Ca^2+^ influx, shown as **E**) average cytosolic Ca^2+^ response, **F**) peak relative Ca^2+^ influx (peak 2) and **G**) ratio of Ca^2+^ influx amplitude (peak 2) divided by AUC (t = 0–705 s). Values show mean ± S.D. for nine wells from three independent experiments. * *P*<0.05 (unpaired t-test).

**Figure 3:**
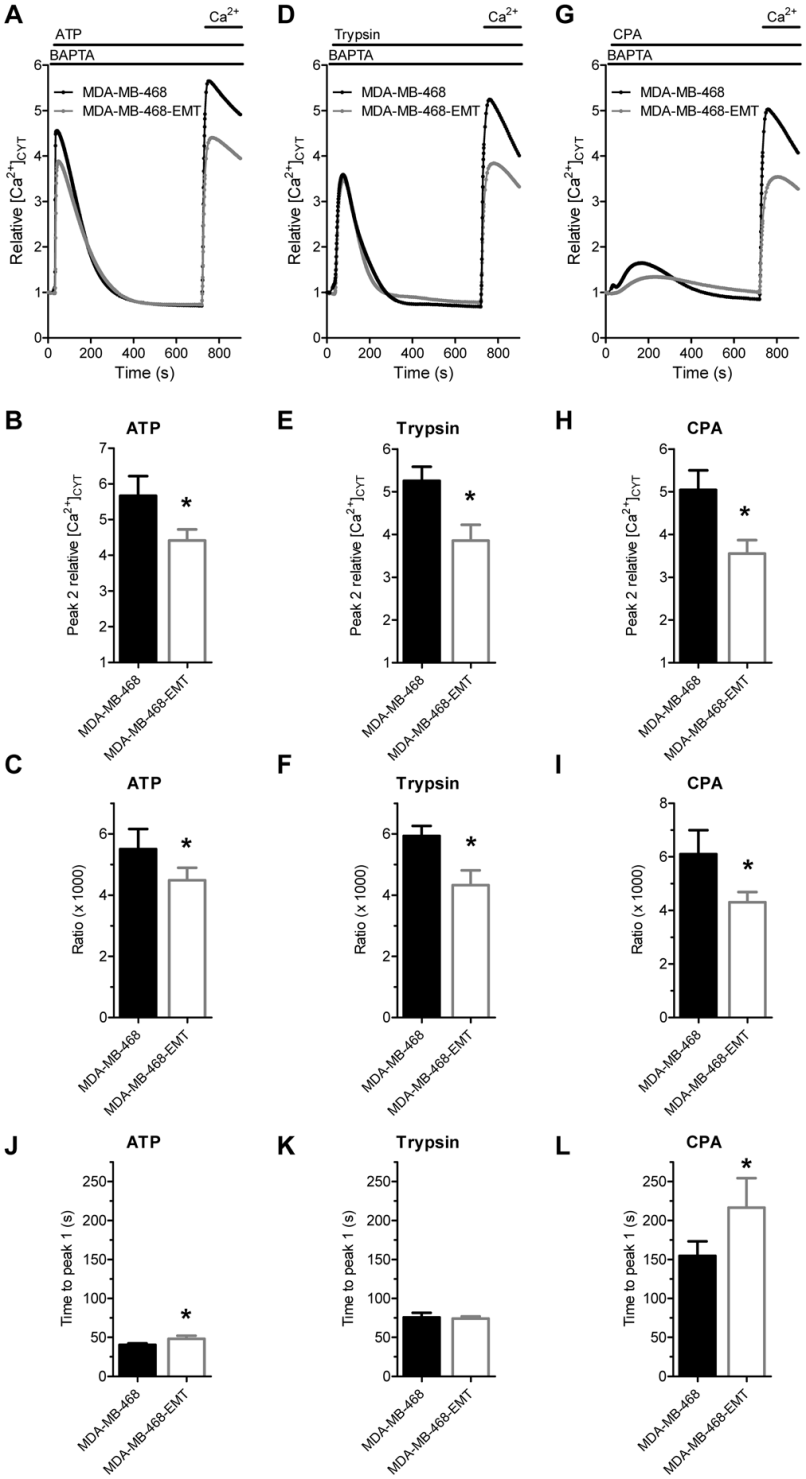
Agonist-stimulated and store-operated Ca^2+^ entry in MDA-MB-468 cells with EGF-induced EMT. Average cytosolic Ca^2+^ response, peak relative Ca^2+^ influx (peak 2) and ratio of Ca^2+^ influx amplitude (peak 2) divided by AUC (t = 0–705 s) mediated by 100 µM ATP (**A–C**), 30 nM trypsin (**D–F**) and 10 µM CPA (**G–I**). Average time to peak (t = 0–705 s) for **J**) ATP, **K**) trypsin and **L**) CPA. Values show mean ± S.D. for nine wells from three independent experiments; * *P*<0.05 (unpaired t-test).

Global cytosolic Ca^2+^ responses in MDA-MB-468 and MDA-MB-231 cells were assessed using a fluorometric imaging plate reader (FLIPR^TETRA^, Molecular Devices). MDA-MB-468 breast cancer cells were seeded at 3×10^4^ cells/well in 96-well microplates (Corning Costar). Cells were serum-deprived (0.5% FBS, 24 h) (Fig. 1, 2 & 3), and treated ± EGF (50 ng/mL, 24 h) as indicated (Fig. 2 & 3) [19], [22]. For Ca^2+^ assays in siRNA-transfected MDA-MB-468 cells a seeding density of 1.5×10^4^ cells/well was used and 4×10^3^ cells/well for MDA-MB-231 cells. Cells were loaded for 1 h at 37°C with 2 µM Fluo-4 AM Ca^2+^ indicator in a solution containing 500 µM probenecid and 5% (v/v) PBX Signal Enhancer (BD Biosciences) in physiological salt solution (PSS; 5.9 mM KCl, 1.4 mM MgCl_2_, 10 mM HEPES, 1.2 mM NaH_2_PO_4_, 5 mM NaHCO_3_, 140 mM NaCl, 11.5 mM glucose and 1.8 mM CaCl_2_) [21]. Cells were allowed to equilibrate to room temperature (15 min) and loading solution was then replaced with a solution containing 500 µM probenecid and 5% (v/v) PBX Signal Enhancer in nominally Ca^2+^-free PSS. Intracellular Ca^2+^ measurements were performed with 470/95 and 515/75 nm excitation and emission filters. Data analysis was performed using ScreenWorks Software (v2.0.0.27, Molecular Devices). The ratio of influx to store release was calculated as follows: [peak 2 amplitude/peak 1 area under the curve (AUC)]*1000. The % reduction in Ca^2+^ influx (e.g., % reduction of non-stimulated Ca^2+^ influx with siORAI1) was calculated by first subtracting the baseline, as follows: [(siNT peak 2 amplitude −1)/(siORAI1 peak 2 amplitude −1)]*100%.

For live cell imaging of Ca^2+^ oscillations, MDA-MB-468 cells were grown to confluency and cells were loaded with Fluo-4 AM as described above. Images were acquired using a Nikon Eclipse TE 300 inverted epifluorescence microscope with a 40× oil objective and 488/550 nm excitation/emission wavelengths. A 33 ms exposure time and 2×2 binning was used; images were acquired every 5 s for approximately 120 cycles prior and subsequent to BAPTA (1.7 mM) addition.

### Real time RT-PCR

For quantitation of changes in gene expression as a consequence of EGF-induced EMT, MDA-MB-468 cells were plated in 6-well plates at seeding density of 8.5×10^4^ cells/well. Cells were serum deprived and treated with EGF (50 ng/mL) for 12 h. Total RNA was isolated using the Qiagen RNeasy Mini Kit. Omniscript RT (Qiagen) was used for reverse transcription and resulting cDNA was amplified using TaqMan Fast Universal PCR Master Mix with TaqMan Gene Expression Assays.

### siRNA transfection

Dharmacon ON-TARGET*plus* SMARTpool™ siRNA was used as per the manufacturer's instructions. This product consists of four rationally designed siRNAs with both sense and anti-sense strand modification to reduce off-target effects [23]. MDA-MB-468 cells were seeded at 1.5×10^4^ cells/well in antibiotic-free media. DharmaFECT4 transfection reagent was used at a concentration of 0.2 µL/well. Knockdown for Ca^2+^ assays and protein isolations was confirmed 48 h post-transfection as per manufacturer's instructions, given that changes in mRNA levels are expected to precede changes in protein expression and functional responses (assessed 96 h post-transfection).

### Immunoblotting

Cell extracts were prepared using protein lysis buffer supplemented with protease and phosphatase inhibitors (Roche Applied Science). Gel electrophoresis was performed under reduced denatured conditions using 4–12% gradient Bis-Tris gels with MOPS running buffer (Invitrogen), and transferred to PVDF membranes. Membranes were blocked for 1 h at room temperature using 5% (w/v) skim milk powder in phosphate buffered saline (PBS) containing 0.1% (v/v) Tween-20. Bands were visualized with chemiluminescence using Super-Signal West Dura substrate (Thermo Fisher Scientific). Image acquisition was performed on a VersaDoc Imaging System (BioRad) and quantitation using ImageJ (v1.45 s, National Institutes of Health), as per the gel analysis method outlined in the ImageJ documentation. Brightness and contrast adjustment were applied uniformly to all gels.

### Cell number and S-phase analysis

MDA-MB-468 cells were seeded in 96-well imaging plates (BD Biosciences) at 5×10^3^ cells/well and treated with siRNA for 96 h. Cells were incubated with EdU (10 mM) for 1 h, fixed with 3.7% (v/v) formaldehyde in PBS and permeabilized with 0.5% (v/v) Triton X-100. Cells were then incubated with the Click-iT reaction cocktail (Alexa Fluor 555; Invitrogen) for 30 min and DAPI (400 nM) for 90 min. Cells were imaged with a 10× objective using the ImageXpress Micro automated epifluorescence microscope (Molecular Devices) based on the following excitation and emission wavelengths: 377/50 and 447/60 nm for DAPI and 531/40 and 593/40 for EdU (Cy3). Cell number and percentage of EdU positive cells was measured using the multi-wavelength cell scoring application module (MetaXpress v3.1.0.83; Molecular Devices).

### Statistical analysis

Statistical analysis was performed using GraphPad Prism (v5.03 for Windows). Statistical tests used in this study are outlined in each figure legend.

## Results

### Non-stimulated calcium influx in MDA-MB-468 breast cancer cells

We assessed agonist-stimulated and store-operated Ca^2+^ entry in MDA-MB-468 breast cancer cells compared to the previously characterized MDA-MB-231 breast cancer cell line [13]. MDA-MB-231 cells are considered more mesenchymal in nature than MDA-MB-468 cells, which (in the absence of EMT inducers) are in a more epithelial state [24]. MDA-MB-468 and MDA-MB-231 cells were treated with ATP (purinergic receptor agonist) or CPA (a sarco/endoplasmic reticulum Ca^2+^ ATPase (SERCA) inhibitor) in nominally Ca^2+^-free conditions, to assess ATP-stimulated Ca^2+^ entry and store-operated Ca^2+^ entry, respectively. ATP and CPA both produced an initial (peak 1) transient increase in cytosolic free Ca^2+^ levels ([Ca^2+^]_CYT_) and Ca^2+^ influx (peak 2) following the re-addition of extracellular Ca^2+^ in MDA-MB-468 (Fig. 1A) and MDA-MB-231 (Fig. 1B) cells. Significant Ca^2+^ influx was observed in the absence of agents to deplete ER Ca^2+^ stores in MDA-MB-468 cells (herein referred to as non-stimulated Ca^2+^ influx; Fig. 1A). Non-stimulated Ca^2+^ influx was greater in MDA-MB-468 cells (approximately 53% of the store-operated Ca^2+^ entry response; Fig. 1A & C) compared to MDA-MB-231 cells (non-stimulated Ca^2+^ influx approximately 3% of the store-operated Ca^2+^ entry response; Fig. 1B & D).

The greater non-stimulated Ca^2+^ influx in MDA-MB-468 breast cancer cells was associated with spontaneous Ca^2+^ oscillations that were attenuated upon removal of non-stimulated Ca^2+^ influx through extracellular Ca^2+^ chelation with BAPTA (Fig. 1E). These spontaneous Ca^2+^ oscillations were asynchronous, as shown by the Y-plane projection through time (Fig. 1F & G) and the representative movie file (Movie S1). Ca^2+^ oscillations appear to be a characterizing feature of MDA-MB-468 breast cancer cells associated with their non-stimulated Ca^2+^ influx. To our knowledge this is the first study to report spontaneous Ca^2+^ oscillations in MDA-MB-468 breast cancer cells and we have not previously seen such spontaneous oscillations in other breast cancer cell lines, however, assessment in MDA-MB-231 breast cancer cells, which had a low level of non-stimulated Ca^2+^ influx, should be conducted and compared to MDA-MB-468 cells.

### EGF-induced EMT attenuates non-stimulated calcium influx in MDA-MB-468 breast cancer cells

To assess possible alterations in non-stimulated Ca^2+^ influx associated with EMT, MDA-MB-468 cells were treated with EGF, a well characterized EMT inducer in this breast cancer cell line. EGF produced significant increases in the EMT markers Twist, Snail, fibronectin and vimentin (Fig. 2A–D). MDA-MB-468 breast cancer cells undergoing EMT (herein referred to as MDA-MB-468-EMT cells) exhibited reduced non-stimulated Ca^2+^ influx (Fig. 2E–G).

### EGF-induced EMT is associated with a reduction in store-operated and agonist-stimulated calcium entry

A reduction in ATP-mediated Ca^2+^ influx (peak 2 amplitude) was observed in MDA-MB-468-EMT cells (Fig. 3A & B). We quantified the ratio of Ca^2+^ influx relative to Ca^2+^ store release (i.e., peak 2 amplitude divided by peak 1 AUC) (Fig. 3C). These results show a reduction in ATP-stimulated Ca^2+^ entry with EGF-induced EMT. Similar reductions in agonist-stimulated Ca^2+^ entry were observed with the protease-activated receptor-2 (PAR-2) activator trypsin (Fig. 3D–F) as well as a reduction in store-operated Ca^2+^ entry as assessed with the SERCA inhibitor CPA (Fig. 3G–I).

To examine the nature of the [Ca^2+^]_CYT_ responses we quantified the time to reach peak [Ca^2+^]_CYT_ levels after ATP, trypsin and CPA addition (Fig. 3J–L). A modest but significant difference in the time to peak was observed with ATP-mediated purinergic receptor activation (40.4±1.9 s versus 48.1±4.0 s; *P*<0.05); this may be explained by altered purinergic-mediated signaling with EGF-induced EMT, as previously reported [19]. No change in the time to reach peak [Ca^2+^]_CYT_ was observed with trypsin-activation. However, a pronounced delay was seen in the time taken to reach peak [Ca^2+^]_CYT_ with CPA treatment in MDA-MB-468 cells after EMT induction (154.7±18.8 s versus 216.6±37.8 s; *P*<0.05). These results are reflective of a substantial change in the nature of the response to CPA, and altered ER Ca^2+^ homeostasis associated with EGF-induced EMT.

### ORAI1 and TRPC1 mRNA levels in MDA-MB-468 cells with EGF-induced EMT

**Figure 4:**
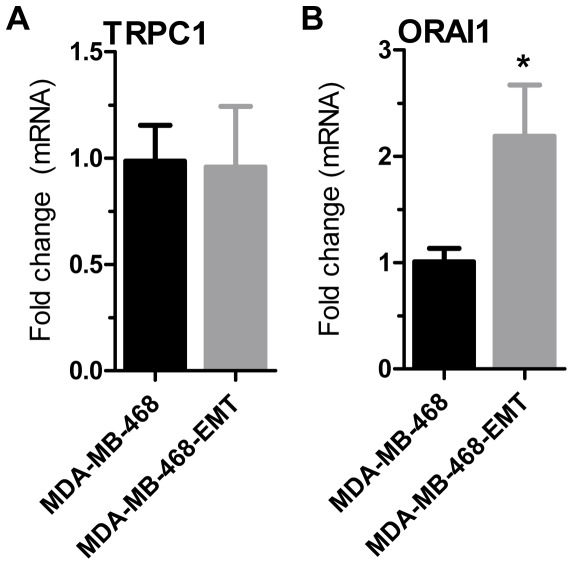
mRNA levels of TRPC1 and ORAI1 with EGF-induced EMT. TRPC1 (**A**) and ORAI1 (**B**) mRNA levels in MDA-MB-468 breast cancer cells stimulated with EGF to induce EMT. Values show mean ± S.D. for nine wells from three independent experiments; * *P*<0.05 (unpaired t-test).

We assessed whether reductions in Ca^2+^ influx with EGF-induced EMT were associated with reduced mRNA levels of TRPC1 and ORAI1 channels. No change in TRPC1 mRNA levels was observed with EGF-induced EMT (Fig. 4A). However, a modest (∼2-fold) increase in ORAI1 mRNA levels (Fig. 4B) was observed. This is consistent with previous studies showing elevated ORAI1 expression with TGFβ-induced EMT in MCF7 cells [20], but does not explain the significantly reduced store-operated Ca^2+^ entry shown in MDA-MB-468-EMT cells.

### ORAI1 and TRPC1 silencing attenuates non-stimulated calcium influx in MDA-MB-468 breast cancer cells

Given that changes in non-stimulated, agonist-stimulated and store-operated Ca^2+^ entry with EGF-induced EMT may reflect changes in ORAI1 or TRPC1 protein expression and/or function, we assessed whether siRNA-mediated inhibition of these two ion channels could phenocopy the changes in Ca^2+^ homeostasis observed with EGF-induced EMT.

**Figure 5:**
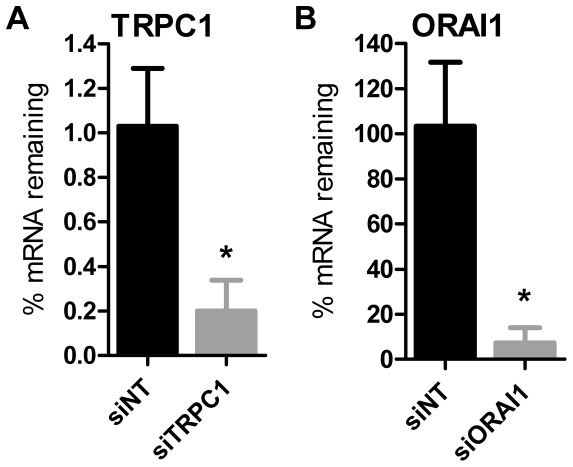
siRNA-mediated silencing of ORAI1 and TRPC1 in MDA-MB-468 breast cancer cells. **A**) Quantitation of TRPC1 mRNA expression 48 h after treatment with TRPC1 siRNA (siTRPC1) relative to the non-targeting siRNA control (siNT). **B**) Assessment of ORAI1 expression 48 h post transfection with ORAI1 siRNA (siORAI1). Values show mean ± S.D. for six wells from three independent experiments; * *P*<0.05 (unpaired t-test).

**Figure 6:**
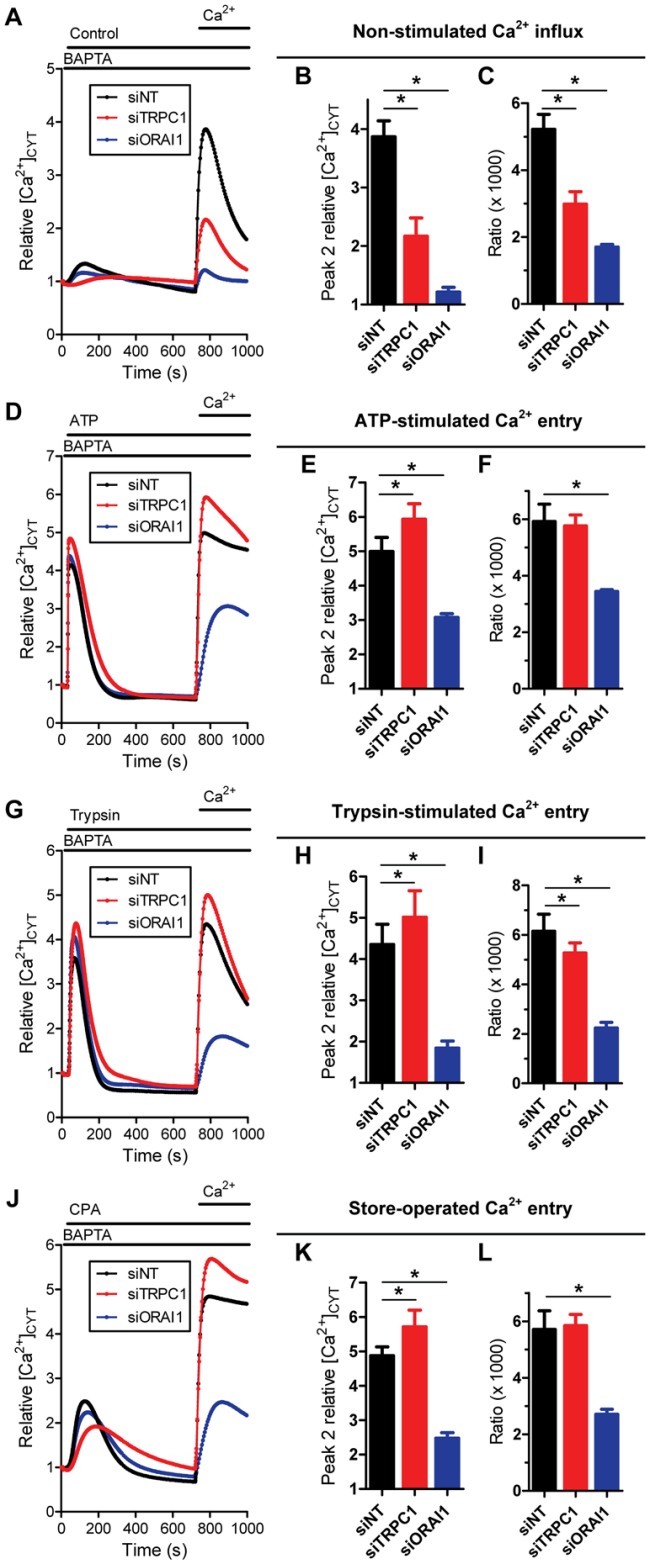
Effect of TRPC1 and ORAI1 silencing on Ca^2+^ influx pathways in MDA-MB-468 breast cancer cells. Non-stimulated Ca^2+^ influx was assessed in MDA-MB-468 breast cancer cells with ORAI1 silencing (siORAI1) or TRPC1 silencing (siTRPC1); **A**) average cytosolic Ca^2+^ response, **B**) peak relative Ca^2+^ influx (peak 2) and **C**) ratio of Ca^2+^ influx amplitude (peak 2) divided by AUC (t = 0-705 s). The role of ORAI1 and TRPC1 in agonist-stimulated Ca^2+^ entry, mediated by 100 µM ATP (**D–F**) and 30 nM trypsin (**G–I**), and store-operated Ca^2+^ entry with 10 µM CPA (**J–L**) was assessed. Data show mean ± S.D. for nine wells from three independent experiments; * *P*<0.05 (one-way ANOVA with Bonferroni post-tests).

Silencing of both ORAI1 (siORAI1) and TRPC1 (siTRPC1) (Fig. 5) inhibited non-stimulated Ca^2+^ influx compared to the non-targeting control (siNT) in MDA-MB-468 cells (Fig. 6A–C). These results demonstrate a role for both of these channels in non-stimulated Ca^2+^ influx in MDA-MB-468 cells.

### Consequences of ORAI1 and TRPC1 silencing on agonist-stimulated and store-operated calcium entry

Silencing of ORAI1 inhibited ATP-stimulated Ca^2+^ entry in MDA-MB-468 breast cancer cells (Fig. 6D). Quantitation of Ca^2+^ influx (peak 2 relative [Ca^2+^]_CYT_ response) (Fig. 6E) and the ratio of peaks (Fig. 6F) showed significant inhibition of ATP-stimulated Ca^2+^ entry with siORAI1. Silencing of TRPC1 produced a modest but significant increase in Ca^2+^ influx (peak 2 relative [Ca^2+^]_CYT_ response), however, this effect was associated with increased ATP-mediated Ca^2+^ store release (peak 1) and so was not associated with alterations in agonist-stimulated Ca^2+^ entry as assessed by the peak ratio (Fig. 6F). Similar to ATP, agonist-stimulated Ca^2+^ entry mediated by trypsin was greatly attenuated by ORAI1 siRNA (Fig. 6G–I). TRPC1 silencing augmented the peak 2 [Ca^2+^]_CYT_ response (Fig. 6H); this was associated with a modest but significant decrease in trypsin-stimulated Ca^2+^ entry as assessed by the peak ratio (Fig. 6I). Only ORAI1 siRNA inhibited store-operated Ca^2+^ entry mediated by Ca^2+^ store-depletion using CPA (Fig. 6J–L). These results suggest that activation of some receptors (e.g., PAR-2) may recruit both ORAI1 and TRPC1 (although to a far lesser extent) to replenish depleted stores in MDA-MB-468 breast cancer cells, whereas other mechanisms are specific for ORAI1 over TRPC1, such as ATP and store-depletion as a consequence of SERCA inhibition.

### Silencing of TRPC1 changes the nature of the [Ca^2+^]_CYT_ response associated with SERCA inhibition

**Figure 7:**
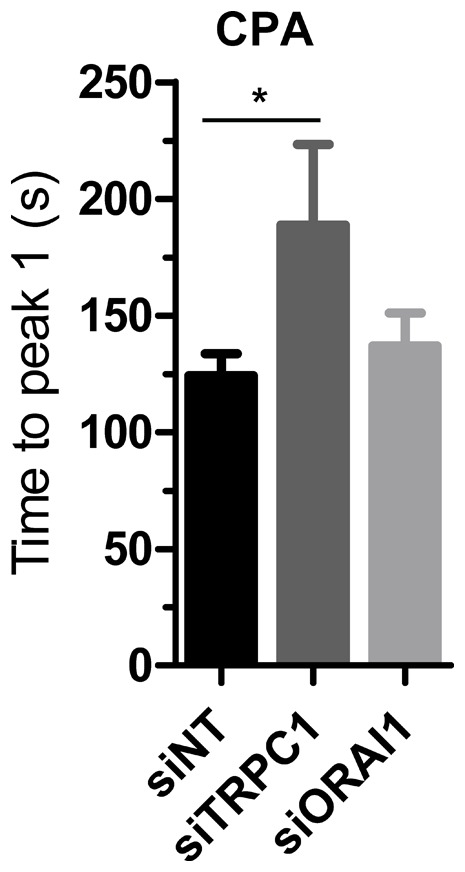
Altered ER Ca^2+^ release kinetics with TRPC1 silencing. Quantitation of the time to reach peak cytosolic Ca^2+^ (t = 0–705 s) with CPA in MDA-MB-468 cells with ORAI1 or TRPC1 silencing. Values show mean ± S.D. for nine wells from three independent experiments; * *P*<0.05 (one-way ANOVA with Bonferroni post-tests).

MDA-MB-468 cells with TRPC1 knockdown exhibited a delay in the time to reach peak [Ca^2+^]_CYT_ with CPA stimulation (Fig. 6J & Fig. 7). This effect was remarkably similar to the change in CPA response observed during EGF-induced EMT (Fig. 3G & L). In contrast ORAI1 silencing did not alter the nature of the CPA response in these cells. The change in Ca^2+^ leak from the ER upon SERCA inhibition in MDA-MB-468 cells with TRPC1 silencing suggests that TRPC1 is a direct regulator of Ca^2+^ homeostasis in the ER.

### TRPC1 silencing inhibits constitutively active ERK1/2 and leads to S-phase reduction

**Figure 8:**
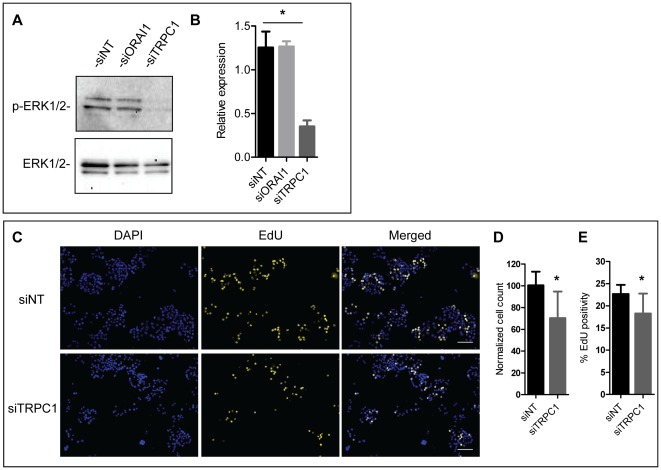
Effect of TRPC1 and ORAI1 silencing on constitutive ERK1/2 activity and cell proliferation. **A**) Representative immunoblot showing constitutive phosphorylation and total expression of ERK1/2 in MDA-MB-468 cells with ORAI1 or TRPC1 silencing. **B**) Densitometric data was obtained from the pooled data (three independent immunoblots) and shows ERK1/2 phosphorylation relative to total ERK1/2 expression; * *P*<0.05 (one-way ANOVA with Bonferroni post-tests). **C**) DAPI (cell number) and EdU staining (showing cells in S-phase of the cell cycle). **D**) Quantitation of the average cell count and (**E**) EdU positivity for nine wells from three independent experiments.* *P*<0.05 (unpaired t-test). All graphs show mean ± S.D. Scale bar represents 100 µm.

As ORAI1 [11] and TRPC1 [25] regulate ERK1/2 activity in other cell types we assessed constitutive ERK1/2 phosphorylation in MDA-MB-468 breast cancer cells with ORAI1 or TRPC1 silencing. Knockdown of ORAI1 had no effect on constitutive ERK1/2 activity in these cells; however, silencing of TRPC1 channels led to a pronounced reduction in constitutively active ERK1/2 in MDA-MB-468 breast cancer cells (Fig. 8A & B).

Sustained ERK1/2 phosphorylation can regulate S-phase entry [26]. Therefore, we assessed whether TRPC1-mediated inhibition of constitutive ERK1/2 activity in MDA-MB-468 breast cancer cells led to reduced cell count and S-phase reduction. Compared to the non-targeting siRNA control, MDA-MB-468 breast cancer cells with TRPC1 silencing showed modest but significant reductions in cell number (Fig. 8C-DAPI and Fig. 8D) and a reduced percentage of cells in the S-phase of the cell cycle (Fig. 8C-EdU and Fig. 8E). Together our findings indicate that TRPC1 silencing in MDA-MB-468 cells reduces the pool of constitutively active ERK1/2 and reduces cell proliferation.

## Discussion

Altered Ca^2+^ influx is a feature of many epithelial cancers, including breast, prostate and ovarian cancer, and may regulate processes important for carcinogenesis [27], [28]. Examples of the significance of Ca^2+^ influx in cancer include the up-regulation of TRPC3 channels in human ovarian cancers, and the ability of TRPC3 knockdown to inhibit growth factor-mediated Ca^2+^ signaling and cell cycle progression in SKOV3 ovarian cancer cells [29]. Molecular components of the store-operated Ca^2+^ entry pathway also regulate cell signaling events important in carcinogenesis, the best characterized of which is ORAI1-mediated activation of the Ca^2+^-dependent transcription factor NFAT [30]. Recent studies show that regions of the Ca^2+^ pump SPCA2 can activate ORAI1 and promote constitutive Ca^2+^ influx and phospho-protein signaling in MCF7 breast cancer cells, independently of Ca^2+^ store depletion [11]. Here we characterized Ca^2+^ influx, in particular mechanisms important for store-operated Ca^2+^ entry, during EGF-mediated EMT in human breast cancer cells and the downstream signaling events regulated by these Ca^2+^ influx pathways. Table 1 summarizes changes in Ca^2+^ homeostasis characterized in this study.

**Table 1:** Summary of changes in Ca^2+^ homeostasis with EGF-induced EMT and silencing of ORAI1 (siORAI1) or TRPC1 (siTRPC1).

	Epithelial	Mesenchymal	siORAI1	siTRPC1
Non-stimulated Ca^2+^ influx	↑	↓	↓	↓
Agonist-stimulated Ca^2+^ entry	↑	↓	↓	↔ or ↓
Store-operated Ca^2+^ entry	↑	↓	↓	↔
ER Ca^2+^ release	Fast	Slow	↔	Slow

Arrows signify whether the epithelial, mesenchymal or knockdown phenotypes are associated with a significant increase (↑) or decrease (↓) or no change (↔) in Ca^2+^ influx via each pathway. Changes in the release of ER Ca^2+^ upon SERCA inhibition are described as fast, slow or unchanged (↔) for each condition.

Ca^2+^ influx following Ca^2+^ store depletion mediated by either agonist-stimulation (with ATP) or via inhibition of SERCA activity (with CPA) was assessed in two phenotypically distinct breast cancer cell lines—MDA-MB-468 and MDA-MB-231. Both cell lines demonstrated Ca^2+^ influx via ATP-stimulated and store-operated Ca^2+^ entry. Surprisingly MDA-MB-468 breast cancer cells also showed pronounced Ca^2+^ influx in the absence of agents to deplete ER Ca^2+^ reserves. This non-stimulated Ca^2+^ influx is not a universal characteristic of breast cancer cell lines [13], and may reflect a proclivity of MDA-MB-468 cells to undergo EMT. MDA-MB-468-EMT cells, characterized by elevated expression of a panel of mesenchymal markers, showed a reduced level of non-stimulated Ca^2+^ influx. These results suggest that in addition to a remodeling of purinergic receptor-mediated Ca^2+^ signaling [19], EGF-induced EMT in MDA-MB-468 breast cancer cells is associated with a significant reduction in non-stimulated Ca^2+^ influx. EGF-induced EMT in MDA-MB-468 breast cancer cells was also associated with reduced agonist-stimulated and store-operated Ca^2+^ entry, in contrast with a recent study by Hu et al. [20], showing elevated store-operated Ca^2+^ entry with TGFβ-induced EMT in MCF7 breast cancer cells. Further studies are therefore required to determine how store-operated Ca^2+^ entry changes in other cancer cell lines and using other methods to induce EMT, for example hypoxia or via the expression of EMT-inducing transcription factors [31], [32].

An array of Ca^2+^-permeable ion channels are implicated in constitutive Ca^2+^ influx, including TRP channels with reported basal activity (e.g., TRPV6) [33], [34] and ORAI channels [11]. Here we show that in MDA-MB-468 breast cancer cells, both ORAI1 and TRPC1 silencing reduce non-stimulated Ca^2+^ influx; suggesting that altered activity of these channels may be a feature of EGF-induced EMT. Mechanistically, the many activators of ORAI1, including STIM1 and −2 [8], [9], [35], [36], and SPCA2 [11], may contribute to an ORAI1-mediated reduction in non-stimulated Ca^2+^ influx. Reduced non-stimulated influx via TRPC1 may also arise through multiple mechanisms such as altered gene expression, channel gating [37], localization [38] or heteromulterization [39], [40]. A transcriptional down-regulation of ORAI1 or TRPC1 does not appear to be involved, as no significant reduction in ORAI1 or TRPC1 expression was observed in MDA-MB-468 cells with EGF-induced EMT. Further studies are required to determine how ORAI1 and TRPC1 may be altered with EGF-induced EMT and to investigate possible changes in other Ca^2+^ influx pathways during this phenotypic switch.

In addition to these channel-specific mechanisms of regulation, control of non-stimulated Ca^2+^ influx may depend on a complex interplay between TRPC1 and ORAI1 channels. The 93% inhibition of non-stimulated Ca^2+^ influx by ORAI1 knockdown would suggest that other plasma membrane Ca^2+^ channels make up only a small (∼7%) component of non-stimulated Ca^2+^ influx. However, TRPC1 knockdown inhibited non-stimulated Ca^2+^ influx by more than 50%. These results suggest that TRPC1 and ORAI1 cooperate to regulate non-stimulated Ca^2+^ influx, and that TRPC1-mediated regulation of non-stimulated Ca^2+^ influx is dependent on Ca^2+^ influx through ORAI1 channels.

Unlike ORAI1 knockdown, silencing of TRPC1 was associated with significant delays in the time to reach peak [Ca^2+^]_CYT_ after SERCA inhibition, providing some evidence for a role for TRPC1 in the regulation of resting ER Ca^2+^. Such a result can be explained by the cellular expression of TRPC1 at sites other than the plasma membrane. A non-plasmalemmal distribution for TRPC1 has been shown in human salivary gland cells [41]. Furthermore, TRPC1 channels have been shown to localize to the sarcoplasmic reticulum (SR) of muscle fibres and regulate SR Ca^2+^ leak [42], and mis-localization of TRPC1 to the ER can occur when TRPC1 is overexpressed in epithelial cells [39], [40], [43]. The existence of functional intracellular TRPC1 channels on the ER is still controversial, owing to the lack of specific TRPC1 antibodies [44], [45] and inherent problems related to channel localization in overexpression systems. However, ER-resident TRPC1 Ca^2+^ channels may promote ER Ca^2+^ leak, similar to the increased Ca^2+^ leak reported with ER-localized TRPP2 Ca^2+^ channels in kidney cells [46], and ER Ca^2+^ leak regulated by Bcl-2 [47], [48]. Mislocalization of TRP channels is also seen in cancer cells [49], [50], with the expression of functional TRPM8 channels on the ER of prostate cancer cells [50]. TRPC1-mediated ER Ca^2+^ leak would trigger Ca^2+^ influx via ORAI1 to replenish lower ER Ca^2+^ reserves. Therefore, the non-stimulated Ca^2+^ influx observed in MDA-MB-468 breast cancer cells, which is sensitive to both TRPC1 and ORAI1 knockdown, may be due to enhanced ER Ca^2+^ leak through TRPC1, the consequence of which is increased store-regulated Ca^2+^ influx through ORAI1.

Ca^2+^ entry following depletion of ER Ca^2+^ stores with CPA (SERCA inhibitor) or following activation of PAR-2 or purinergic receptors was greatly inhibited in cells with ORAI1 silencing. While a critical role for ORAI1 in store-operated Ca^2+^ entry is widely accepted, the role of TRPC1 in this process is controversial [4], [41], [43], [51]. Although in our study TRPC1 knockdown produced modest reductions in trypsin-stimulated Ca^2+^ entry, the absence of pronounced changes in Ca^2+^ influx following store depletion with CPA indicates that TRPC1 is not a regulator of store-operated Ca^2+^ entry in MDA-MB-468 breast cancer cells under these conditions. These findings are consistent with studies in vascular smooth muscle cells, where silencing of TRPC1 had no effect on store-operated Ca^2+^ entry [18].

Rather than acting as a store-operated Ca^2+^ entry channel *per se*, we provide evidence for a model in MDA-MB-468 cells whereby TRPC1-indirectly activates Ca^2+^ influx via ORAI1 in a highly contextual manner. Basal ER Ca^2+^ leak, which could be in-part mediated by ER-localized TRPC1, would create a requirement for Ca^2+^ influx mediated by ORAI in MDA-MB-468 cells (non-stimulated Ca^2+^ influx). However, under conditions that produce large reductions in ER Ca^2+^ (e.g., SERCA inhibition with extracellular Ca^2+^ chelation), the contribution of TRPC1 to Ca^2+^ influx may be masked by maximal ORAI1 activation.

Differences in the functional consequences of ORAI1 and TRPC1 knockdown in MDA-MB-468 cells were also reflected in effects on constitutive ERK1/2 phosphorylation. Knockdown of ORAI1 in MDA-MB-468 breast cancer cells had no effect on the level of constitutively active ERK1/2. This is in contrast to MCF7 breast cancer cells, where ORAI1 silencing almost completely abolishes constitutive ERK1/2 activity [11]. Differences in the regulation of ERK1/2 between breast cancer cell lines is not unexpected given the cell-type and context-specific nature of this signal; its spatiotemporal regulation; and the complexity of upstream phospho-protein regulation [52]. In MDA-MB-468 breast cancer cells, silencing of TRPC1 was associated with a marked reduction in constitutive ERK1/2 activity. Differential regulation of ERK1/2 by TRPC1 (but not ORAI1) suggests that constitutive ERK1/2 signaling in MDA-MB-468 cells is not regulated by global increases in [Ca^2+^]_CYT_, as both agents reduced non-stimulated Ca^2+^ influx. Given that both ERK and upstream Ras-GTPases may localize to the ER membrane [53], [54], regulation of constitutive ERK1/2 signaling in MDA-MB-468 cells may be dependent on TRPC1 channels also located on the ER membrane and/or the nature of the Ca^2+^ signal regulated by TRPC1.

To explore the possible consequences of TRPC1-mediated reductions in ERK1/2 signaling, we assessed cell proliferation in MDA-MB-468 breast cancer cells with TRPC1 silencing. Recent studies show that sustained (but not transient) ERK1/2 activation is a regulator of S-phase entry in NIH3T3 mouse embryonic fibroblasts [26]. MDA-MB-468 breast cancer cells with TRPC1 silencing showed reduced cell number and the percentage of cells in the S-phase of the cell cycle. These results indicate that Ca^2+^ signaling via TRPC1 regulates a constitutive ERK1/2 phosphorylation and cell proliferation in MDA-MB-468 cells breast cancer cells.

The current study has characterized mechanisms regulating Ca^2+^ influx in MDA-MB-468 human breast cancer cells and assessed alterations in Ca^2+^ signaling as a consequence of EGF-induced EMT. MDA-MB-468 breast cancer cells in the epithelial state have a high degree of non-stimulated Ca^2+^ influx through ORAI1, potentially due to alterations in ER Ca^2+^ reserves. Our studies suggest that some of the disparities in the reported roles for TRPC1 in store-operated Ca^2+^ entry may be explained by the context-specific signaling of these channels (i.e., cellular localization, the degree of Ca^2+^ store depletion and non-stimulated Ca^2+^ influx); factors which are likely to vary between cell types and experimental conditions. Our studies also define a specific role for TRPC1 in the regulation of the constitutively active pool of ERK1/2 in MDA-MB-468 cells. Given the potential for Ca^2+^ to regulate processes important in carcinogenesis [28], and the identification of TRPC1 as a regulator of protein kinases, it is now imperative to establish the cellular localization of Ca^2+^ channels in cancer cells, and the molecular mechanisms governing their cellular distribution.

## Supporting Information

Movie S1 **Ca^2+^ oscillations in MDA-MB-468 breast cancer cells.** Relates to Fig. 1 E–G; representative of at least five movies from three independent experiments. Movie is shown at 75× speed.
(AVI)
